# Reactive Astrocytes: Critical Players in the Development of Chronic Pain

**DOI:** 10.3389/fpsyt.2021.682056

**Published:** 2021-05-28

**Authors:** James Tang, Mercedes Bair, Giannina Descalzi

**Affiliations:** Department of Biomedical Sciences, Ontario Veterinary College, University of Guelph, Guelph, ON, Canada

**Keywords:** chronic pain, plasticity, astrocytes, cytokine, TNFα, lactate, gliotransmission

## Abstract

Chronic pain is associated with long term plasticity of nociceptive pathways in the central nervous system. Astrocytes can profoundly affect synaptic function and increasing evidence has highlighted how altered astrocyte activity may contribute to the pathogenesis of chronic pain. In response to injury, astrocytes undergo a shift in form and function known as reactive astrogliosis, which affects their release of cytokines and gliotransmitters. These neuromodulatory substances have been implicated in driving the persistent changes in central nociceptive activity. Astrocytes also release lactate which neurons can use to produce energy during synaptic plasticity. Furthermore, recent research has provided insight into lactate's emerging role as a signaling molecule in the central nervous system, which may be involved in directly modulating neuronal and astrocytic activity. In this review, we present evidence for the involvement of astrocyte-derived tumor necrosis factor alpha in pain-associated plasticity, in addition to research suggesting the potential involvement of gliotransmitters D-serine and adenosine-5′-triphosphate. We also discuss work implicating astrocyte-neuron metabolic coupling, and the possible role of lactate, which has been sparsely studied in the context of chronic pain, in supporting pathological changes in central nociceptive activity.

## Introduction

Chronic pain is associated with long lasting structural and functional reorganization of nociceptive circuits in the spinal cord and brain (1, 2). Historically considered supportive cells, mounting evidence indicates that astrocytes are dynamic players in neuroplasticity, and astrocytes have become increasingly recognized as active players in synaptic changes associated with chronic pain states. Pathologies of the central nervous system (CNS) often involve reactive astrogliosis, a process whereby astrocytes undergo a shift in morphology and function (3, 4). Reactive astrogliosis is associated with astrocyte hypertrophy, upregulated expression of glial fibrillary acidic protein (GFAP), and altered gene expression (5). Repeatedly, studies utilizing various rodent models of chronic pain show that GFAP is upregulated in the spinal cord (6–11) and brain areas involved in processing the sensory and affective components of pain, including the anterior cingulate cortex (ACC) (12–16), somatosensory cortex (17), amygdala (18, 19), thalamus (20), and ventrolateral periaqueductal gray (21–24). Notably, inhibiting astrocyte activity in the spinal cord (25–27) and primary somatosensory cortex (17) has been shown to reduce pain hypersensitivity. Chronic pain is also often associated with depression and elevated anxiety, and astrocyte inhibitors administered into the ACC have been shown to alleviate anxiety and depression-related symptoms in rodents (13, 15). Moreover, recent research has provided evidence for glial activation in the spinal cord and brain of patients with various chronic pain syndromes (28–31), and enhanced astrocyte activation has been observed in the spinal dorsal horn of HIV patients with chronic pain (32). Given the consistent theme of astrocyte activation, recent research has focused on investigating the role of reactive astrocytes in the pathogenesis of chronic pain. This brief review will cover recent literature identifying astrocyte-derived cytokines, gliotransmission, and altered astrocyte-neuron metabolic coupling as potential contributors to the persistently altered synaptic activity observed in chronic pain states.

## Cytokines

Cytokines are important regulators of inflammatory responses, and the activity of several pro-inflammatory and anti-inflammatory cytokines within the peripheral and central nervous systems (PNS and CNS, respectively) have been found to correspond with chronic pain states (33). Astrocytes and microglia release and respond to cytokines and play a significant role in immune responses of the CNS. Substantial research has uncovered microglia-mediated cytokine activity in chronic pain states. For example, spinal microglia are activated in inflammatory and neuropathic pain models (34, 35), and are associated with the production of pro-inflammatory cytokines such as tumor necrosis factor alpha (TNFα), interleukin-1 beta (IL-1β), IL-6, and interferon gamma (IFNγ) (34, 36). Additionally, specific microglia inhibitors can attenuate pain-related behaviors while significantly inhibiting the upregulation of pro-inflammatory cytokines (37, 38). But while microglia contribute to the pro-inflammatory phenotype in concert with astrocytic activity, the present review will focus specifically on astrocytes [for excellent reviews regarding microglia in chronic pain, see references (39) and (40)]. Numerous *in-vitro* observations show that stimuli ranging from lipopolysaccharide, metabolic and mechanical stress, and neurotropic viruses, stimulate astrocytic production and release of cytokines (41–45). These include, but are not limited to, TNFα, IL-1α, IL-1β, IL-6, IFNα, IFN-β, and IFNγ (41–45). Work done in post-mortem tissue from chronic pain patients has associated spinal astrocyte activation with production of inflammatory cytokines such as IL-1β and TNFα (32), while an *in vivo* pain model has shown that inhibiting astrocytes with the toxin L-α-aminoadipate can reduce IL-1β expression and mechanical allodynia (46). Notably, elevated levels of pro-inflammatory cytokines have been observed in the blood and cerebral spinal fluid of patients with chronic pain, and have been shown to positively correlate with subjective ratings of pain intensity (47, 48).

## TNFα Directly Modulates Nociceptive Neuronal Activity

TNFα's pathogenicity is well-documented in the peripheral nervous system. Direct injection of TNFα into the sciatic nerve or acute application to the L4 dorsal root ganglion (DRG) induces signs of mechanical allodynia and thermal hyperalgesia, however symptoms appear to be short-lived with recovery occurring within a couple days (49–51). Chronic application of a pad soaked in TNFα to the L5 nerve root or chronic perfusion of the DRG resulted in symptoms persisting beyond 7 days (52, 53), suggesting a requirement for extended exposure in the periphery to initiate long lasting pain. TNFα perfusion at the DRG is also able to enhance pain symptoms associated with compression of the DRG (53).

In the central nervous system, chronic pain induction elevates TNFα levels in the dorsal horn of the spinal cord, which either coincide with or are temporally close to the onset of mechanical and thermal hypersensitivity (54–58). In the brain, the time course of TNFα elevations in chronic pain models varies between different regions. For example, TNFα levels are elevated in the locus coeruleus prior to the onset of chronic constriction injury induced thermal hyperalgesia, while rises and falls in hippocampal TNFα approximately correspond to symptom onset and dissipation, respectively (58, 59). In the ACC, TNFα expression increases shortly after the onset of spared nerve injury-induced mechanical allodynia (60).

**Figure 1:**
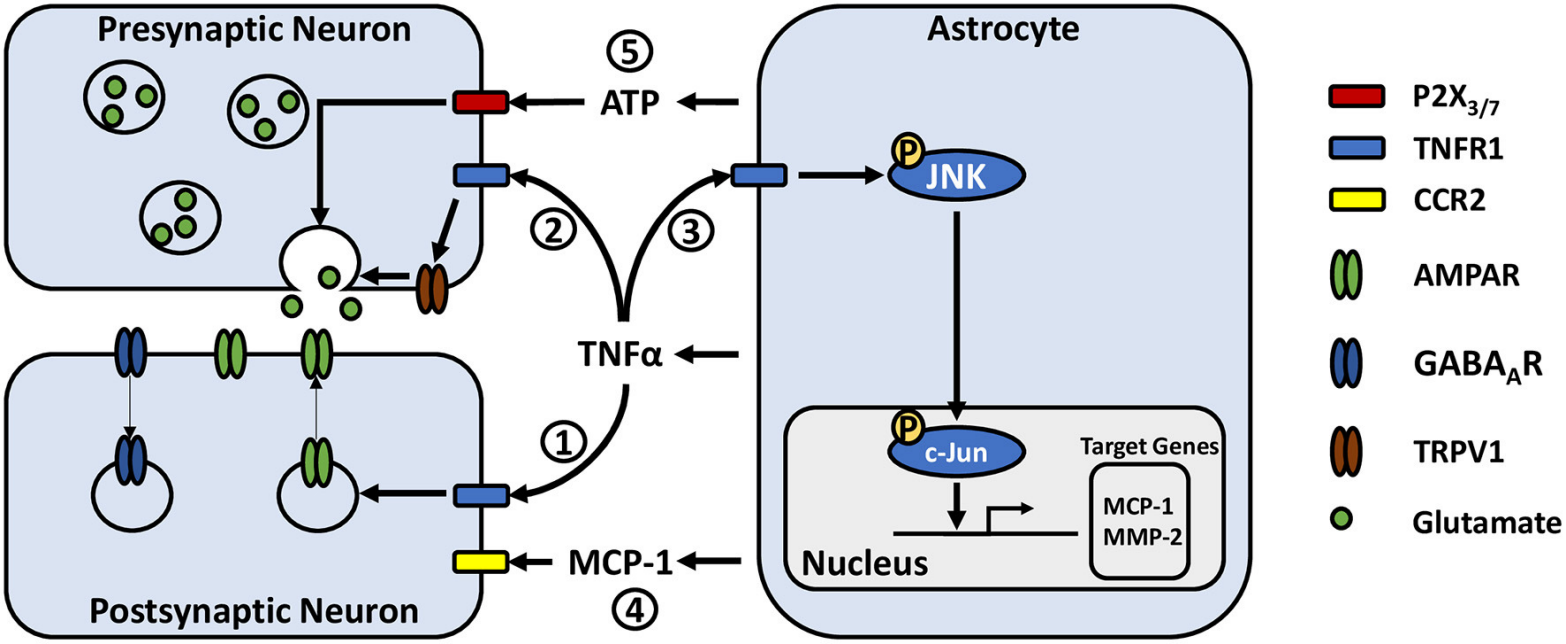
Potential astrocyte-neuron signaling pathways in modulating pain-related synaptic transmission. (1) TNFα acts on neuronal TNFR1 resulting in rapid trafficking of GluR2-lacking AMPA receptors to the postsynaptic membrane and internalization of postsynaptic GABA_A_ receptors. (2) TNFα increases presynaptic glutamate release, potentially by activating or increasing the expression of transient receptor potential subtype V1. (3) TNFα acts on astrocytic TNFR1, inducing phosphorylation of JNK. JNK phosphorylates c-Jun, which dimerizes with c-Fos to form the AP-1 transcription factor, leading to transcription of target genes such as MCP-1 and MMP-2. (4) MCP-1 is released from astrocytes where it can act on neurons via CCR2, modulating their excitability. (5) ATP released from astrocytes may act on pre-synaptic neuronal P2X_3_ and P2X_7_ receptors, stimulating glutamate release.

Research has shown that TNFα is able to modulate synaptic activity by acting directly on neurons via tumor necrosis factor receptor 1 (TNFR1) (61, 62) (Figure 1). Notably, astrocyte derived TNFα increases AMPA receptor and decreases GABA_A_ receptor surface expression in cultured hippocampal neurons leading to increases in frequency and amplitude of mini excitatory postsynaptic currents (mEPSCs), and decreases in amplitude of mini inhibitory postsynaptic currents (61–63). Similar observations have been made in slices from the ACC, where TNFα increases the amplitude of evoked EPSCs and mEPSC frequency (64). TNFα can also increase the probability of presynaptic neurotransmitter release (64), through mechanisms that may involve the cation channel TRPV1 (65, 66). This TNFα mediated increase in neuronal excitability may participate in homeostatic synaptic scaling resulting from depressed synaptic activity, a process which is reliant on astrocytic rather than neuronal production of TNFα (67). Later work in neuronal cultures argues that rather than simply shifting neurons toward increased excitation, TNFα may act to permit rather than drive synaptic plasticity (68). Indeed, bidirectional effects have been observed, whereby high concentrations of TNFα (1 ug/mL) has been shown to impair the induction of long-term potentiation (LTP), while low concentrations (1 ng/mL) facilitate LTP (69). Studies assessing cytokine concentrations in chronic pain patients have found TNFα levels ranging from a few pg/mL or less in cerebrospinal fluid (47, 70), up to approximately 50 pg/mL in blood (48, 71, 72). As these concentrations are far below the level at which TNFα was found to impair LTP, TNFα release from reactive astrocytes may be more likely to instead facilitate synaptic potentiation. Accordingly, animal models of chronic inflammatory pain have also shown persistent 1–3 pg/mg increases in TNFα above baseline in the ACC and basolateral amygdala, which were associated with enhanced synaptic transmission in these regions, suggesting a possible role of astrocyte-derived TNFα in pain-induced hyperactivity (64, 73).

## TNFα Activates the JNK Signaling Pathway in Astrocytes

Astrocyte-microglia crosstalk via cytokine signaling is emerging as an important mechanism in the development of chronic pain. Microglial release of IL-18 has been associated with astrocyte activation, while astrocytic CXCL12 has been reported to influence microglia, contributing to the development of mechanical allodynia in neuropathic and migraine pain models (74–76). Like neurons, both astrocytes and microglia express TNFR1 (77, 78) and are able to respond to TNFα (Figure 1). Recent cell culture studies have reported that activated microglia may induce reactive astrogliosis through the release of TNFα amongst other cytokines (79, 80). Additionally, astrocytic TNFα has been reported to exert autocrine effects in culture (81). A series of experiments by Yong Jing Gao and colleagues found evidence for a signaling pathway by which TNFα-induced astrocyte activation could contribute to persistent pain hypersensitivity. They showed in primary astrocyte cultures that TNFα induces transient TNFR1-dependent phosphorylation of c-Jun N-terminal kinase 1 (pJNK1) (82), a mitogen-activated protein kinase (MAPK). pJNK1 phosphorylates and activates c-Jun, which is part of the activator protein 1 transcription factor, leading to gene transcription (83). Activation of these astrocytes led to JNK1-dependent production and release of monocyte chemoattractant protein 1 (MCP-1) amongst other chemokines (82). Using *in vivo* approaches, they showed that TNFα injection into the mouse spinal cord induced JNK-dependent mechanical allodynia and thermal hyperalgesia at 3 h with a concomitant increase in MCP-1 expression in astrocytes (82, 84). Additionally, intrathecal injection of astrocytes incubated with TNFα were sufficient to induce MCP-1 dependent mechanical allodynia (84). These results are paralleled with their observations in a spinal nerve ligation model of neuropathic pain, where astrocytic MCP-1 is upregulated, and that pain hypersensitivity is significantly reduced by JNK inhibition and to a lesser degree by MCP-1 inhibition (82).

MCP-1 signaling via CC chemokine receptor type 2 (CCR2) modulates neuronal excitability. In culture, MCP-1 significantly reduces the responsiveness of neurons to GABA, as shown by a decrease in GABA-induced inward currents mediated by GABA_A_ receptors (85). MCP-1 also causes significant increases in parameters indicating neuronal hyper-excitability, including decreased action potential current and voltage thresholds, as well as an increase in number of evoked action potentials (86). In spinal cord slice preparations, MCP-1 bath application dose-dependently enhances the frequency and amplitude of spontaneous EPSCs while enhancing inward AMPA and *N*-methyl-D-aspartate (NMDA) induced currents (82). Given these findings, persistent elevation of MCP-1 by TNFα provides a mechanism by which nociceptive sensitization may be maintained, as a constant shift toward excitation may reduce the threshold for initiating neuronal activity.

In addition to the induction of MCP-1, CCR2, which is constitutively expressed in the spinal dorsal horn (85), is upregulated in chronic pain states further amplifying the effects of MCP-1. In the peripheral nervous system, chronic compression injury to DRG neurons elevates CCR2 expression, increasing their likelihood to depolarize in response to MCP-1 *in vivo* and in dissociated neuronal cultures (86, 87). In bone cancer and trigeminal neuropathic pain models, CCR2 protein expression increases significantly in neurons of the ipsilateral superficial dorsal horn and medullary dorsal horn respectively, coinciding with onset of pain hypersensitivity (88, 89). CCR2 is also elevated in the nucleus accumbens shell where it modulates both depressive and pain-related symptoms (90), and the periaqueductal gray and rostral ventromedial medulla (24) which are involved in modulation of spinal nociceptive pathways (91).

It is likely that MCP-1 is not solely responsible for the nociceptive effects of JNK signaling, as MCP-1 inhibition only partially reduces pain hypersensitivity when compared to JNK inhibition (82). Matrix metalloproteinase-2 (MMP-2) and MMP-9, enzymes involved in degradation of the extracellular matrix [reviewed by Murphy, Nagase (92)], are also regulated by JNK signaling and secreted from astrocytes (93–95), and have been implicated in chronic pain. Work done in the hippocampus, classically associated with learning and memory rather than pain, has found that MMP-9 proteolytic activity contributes to maintenance but not induction of LTP (96), and MMP-9 alone is sufficient to enhance excitatory postsynaptic potentials and dendritic spine volume in CA1 neurons (97). In chronic pain however, while MMP-9 is upregulated in the peripheral nervous system, its expression in the CNS is minimal (98) and appears to be derived from DRG neurons rather than from astrocytes (99).

In contrast to MMP-9, delayed upregulation of MMP-2 is observed in spinal astrocytes following spinal nerve ligation, reaching significance 10 days after surgery (99). MMP-2 is sufficient to induce mechanical allodynia and is associated with cleavage of IL-1β, a major pro-inflammatory cytokine involved in neuroinflammation which works synergistically with TNFα. IL-1β can stimulate additional release of MMP-2 by activating extracellular signal-regulated kinase (ERK) 1/2 in astrocytes (99). Later work has identified the induction of neuronal MMP-2 at an earlier time point, coinciding approximately with symptom onset in a chronic post-ischemia pain model (100). Inhibiting MMP-2 reduces spinal GFAP levels along with decreased phosphorylation of JNK1/2 following induction of chronic pain (100). Thus, elevated neuronal MMP-2 may initially contribute to the induction of astrogliosis in the spinal cord, resulting in the phosphorylation of JNK in astrocytes, and release of both MMP-2 and MCP-1 during the chronic phase of pain. In line with this potential chain of events, there is some evidence suggesting a delay between onset of behavioral symptoms and elevated phosphorylation of JNK1. In two models of neuropathic pain, whereas behavioral symptoms manifested in under a day, increases in pJNK1 levels in the spinal dorsal horn were not observed until day 3 in a spinal nerve ligation model (101) or day 7 in a spared nerve injury model (102), suggesting a potential role for JNK1 in the transition from acute to chronic pain. Accordingly, in a CFA model of chronic inflammatory pain, whereas chronic intrathecal infusion of the JNK1 inhibitor D-JNKI-1 failed to reduce mechanical allodynia during the induction phase, tested at 6 h post CFA injection, it significantly reduced allodynia during the maintenance phase tested days 1–4 post CFA injection (103). In contrast, a study employing a mouse model of chronic post-ischemia pain observed elevations in pJNK1 at the same time as the development of behavioral symptoms (100), and JNK inhibition produced a significant analgesic effect at pain onset, indicating mechanistic differences in chronic pain development resulting from different injuries.

## Gliotransmission in Chronic Pain

Beyond their role in inflammatory signaling, astrocytes can detect neuronal activity through a variety of membrane receptors, inducing intracellular Ca^2+^ responses (104, 105) and subsequent release of neuromodulatory substances, known as gliotransmitters (106–108); these include glutamate, GABA, adenosine-5'-triphosphate (ATP), and D-serine, which bind to an array of pre- and post-synaptic neuronal receptors and influence synaptic transmission (109). The relevance of gliotransmission in chronic pain is currently under debate (110, 111), however emerging evidence indicates a potential role in chronic pain, including findings that gliotransmission is enhanced in reactive astrocytes (112–116) and modulated by inflammatory mediators (117, 118).

D-serine is a potent co-agonist which binds to the glycine site of NMDA receptors (119). It is synthesized by the enzyme serine racemase, which catalyzes the conversion of L-serine to D-serine (120). While serine racemase is primarily expressed in neurons (121), recent evidence shows that reactive astrocytes in traumatic brain injury, Alzheimer's disease, and pain models express the enzyme (115, 116, 122). D-serine released by reactive astrocytes is implicated in the expression of dynamic mechanical allodynia in chronic and acute models of orofacial pain, as well as static allodynia in chronic neuropathic pain (122–125). In these models, degradation of D-serine by D-amino acid oxidase or inhibition of serine racemase by L-serine O-sulfate can prevent the induction of mechanical allodynia, or reduce mechanical allodynia after onset (122–125). Despite neurons being capable of releasing D-serine (126), the finding that astrocyte inhibition reduces mechanical allodynia, which can be reversed by exogenous D-serine further suggests the specific requirement for astrocytes as a source of D-serine (124).

Another gliotransmitter that has been identified as a potential player in chronic pain is ATP. ATP acts primarily through the ionotropic P2X and metabotropic P2Y purinergic receptor families, which are expressed on many cell types in the CNS including neurons, astrocytes, and microglia [for a review see Burnstock (127)]. P2X receptor activation facilitates synaptic transmission by increasing presynaptic glutamate release (128, 129) and inducing EPSCs (130–132), while P2Y receptors primarily mediate inhibitory effects by reducing presynaptic glutamate release (129, 133). However, its functions at excitatory synapses are increasingly observed to be quite complex.

Work by Zhang et al. (134, 135) has provided evidence for the involvement of purinergic signaling on neurons in rats with chronic visceral hypersensitivity, showing that P2X_7_ and P2X_3_ are both upregulated and colocalize with the presynaptic marker synaptophysin in the insular cortex (134, 135). Additionally, inhibitors for either P2X_7_ or P2X_3_ reduced glutamatergic synaptic activity and pain-like symptoms, whereas agonists for either receptor had the opposite effect, elevating synaptic activity and inducing visceral hypersensitivity. Although the cellular source of ATP and whether it was enhanced was not tested in this model, studies by two separate groups in neuropathic pain models have found evidence suggesting that astrocytic ATP release in the spinal cord contributes to pain hypersensitivity. One study by Cui et al. (136) found that inhibiting the mammalian target of rapamycin signaling pathway could reduce ATP release from cultured astrocytes and inhibit neuropathic pain-induced ATP elevation in cerebral spinal fluid. They associated this finding with the analgesic effect of rapamycin (136). Koyanagi et al. (137) investigated diurnal fluctuations in glucocorticoids and its effects on mechanical allodynia and observed that oscillations of plasma corticosterone levels corresponded to the oscillations of spinal ATP and mechanical allodynia. Intrathecal corticosterone injection induced mechanical allodynia which was dependent on microglial P2Y_12_ and also induced ATP release from cultured astrocytes via serum/glucocorticoid regulated kinase 1 signaling (137). Here again like cytokine signaling, ATP signaling involves both astrocytes and microglia and represents another mechanism by which they can interact. Mixed glial culture studies have shown that activation of microglial P2 receptors by either exogenous or astrocyte-derived ATP induces the release of extracellular vesicles, which can in turn modify astrocyte activity (138, 139). Additionally, microglia can release ATP, which has been shown to indirectly modulate excitatory neuronal activity through binding to astrocytic P2Y_1_ receptors in hippocampal slices (113). These findings provide a functional basis which may translate to pain-associated areas in the CNS. Indeed, studies in neuropathic pain models have associated the induction and activity of spinal microglia P2 receptors with the development of mechanical hypersensitivity (140, 141) [for a more in-depth discussion see review by Trang et al. (142)]. Given the variety of cells that can both release and respond to ATP, there is insufficient data to determine whether direct astrocyte to neuron purinergic signaling in the CNS is involved in chronic pain. However, due to the evidence for purinergic signaling in modulating synaptic activity, it remains a possible pathway by which astrocytes can influence maladaptive plasticity in nociceptive circuits of the CNS.

## Altered Glutamate-Glutamine Cycling in Chronic Pain

In addition to the release of neuromodulatory substances, astrocytes are metabolically coupled to neurons. They participate in glutamate clearance by taking up glutamate present at the synaptic cleft, which can then be converted to glutamine via glutamine synthetase (GS) and exported in a process known as the glutamate-glutamine cycle (143). Mounting human and animal data suggest that the development of chronic pain may also involve changes in glutamate-glutamine homeostasis. For example, in healthy human subjects, glutamate and glutamine levels positively correlate with subjective evoked pain ratings in pain-associated areas such as the ACC, mid-cingulate cortex, insula, dorsolateral prefrontal cortex, and thalamus (144, 145). Moreover, elevated combined levels of glutamate and glutamine have been observed in the ACC of patients with a range of chronic pain conditions (146), the thalamus of migraine patients (147), and in the right amygdala of female fibromyalgia patients (148). Notably, glutamate uptake by astrocytes, which is mediated via the glutamate transporters GLT-1 and GLAST (149), appears to show biphasic alterations in rodent models of neuropathic pain. Specifically, within the first 5 days following nerve injury, astrocytic expression of both in GLT-1 and GLAST is upregulated in the ipsilateral spinal dorsal horn (150, 151), which is followed by a prominent decrease in expression below baseline at 7 days post-injury and beyond (150–153). Critically, changes in glutamate uptake may play a causal role in chronic pain development, as inhibiting glutamate transporter upregulation enhances the onset and magnitude of pain-related behaviors (151), whereas transgenic upregulation of spinal GLT-1 can disrupt the induction of, and partially reverse, mechanical and thermal hypersensitivity in neuropathic and inflammatory pain models (154, 155). Interestingly, upregulation of GLT-1 was associated with a decreased number of dorsal horn neurons expressing the immediate early gene ΔFosB, indicating a reduction in neuronal activity (155). Additionally, acute inhibition of spinal GS has also been found to transiently reduce mechanical allodynia in a rat model of chronic pulpitis (156). This was accompanied by a reduction in the enhanced response of wide dynamic range neurons, located in the medullary dorsal horn, to mechanical stimulation (156). There are some discrepancies in the literature, as it has been found for example that intracisternal injection of DL-threo-β-benzyloxyaspartate, an inhibitor of GLT-1, GLAST, and the neuronal glutamate transporter EAAC1, can reduce rather than enhance CFA-induced orofacial heat hyperalgesia (157). Despite this, the evidence suggests that glutamate-glutamine cycling is altered in chronic pain states. It should be noted however, that while GLT-1 and GLAST are predominantly expressed on astrocytes, microglia in the spinal cord and brainstem have been shown to express both transporters following peripheral nerve injury (152, 158) and likely participate in mediating aberrant glutamate dynamics.

## The Astrocyte-Neuron Lactate Shuttle

Astrocyte-neuronal metabolic coupling may also play a critical role in the development of chronic pain. Pain-induced neuroplasticity within spinal and brain regions is believed to promote the transition from acute to chronic pain, and mounting evidence indicates that astrocytes provide neurons with energy in an activity-dependent manner. In particular, astrocytes are the primary sites of glycogen storage in the CNS (159). In response to neuronal activity, astrocytes can rapidly metabolize glycogen to lactate (160) and export it to neurons, where it is converted to pyruvate, and metabolized to ATP via the citric acid cycle and oxidative phosphorylation to serve as a source of energy (161–163). L-lactate derived from astrocytes has been investigated as an energy source for neurons, and altered lactate metabolism is associated with diseases such as Alzheimer's (164–166), epilepsy (167), multiple sclerosis (168, 169), and depression (170). Astrocyte metabolism can also be significantly modified by a variety of cytokines (171). Therefore, it is of interest whether altered astrocytic lactate dynamics are involved in the development and maintenance of chronic pain.

**Figure 2:**
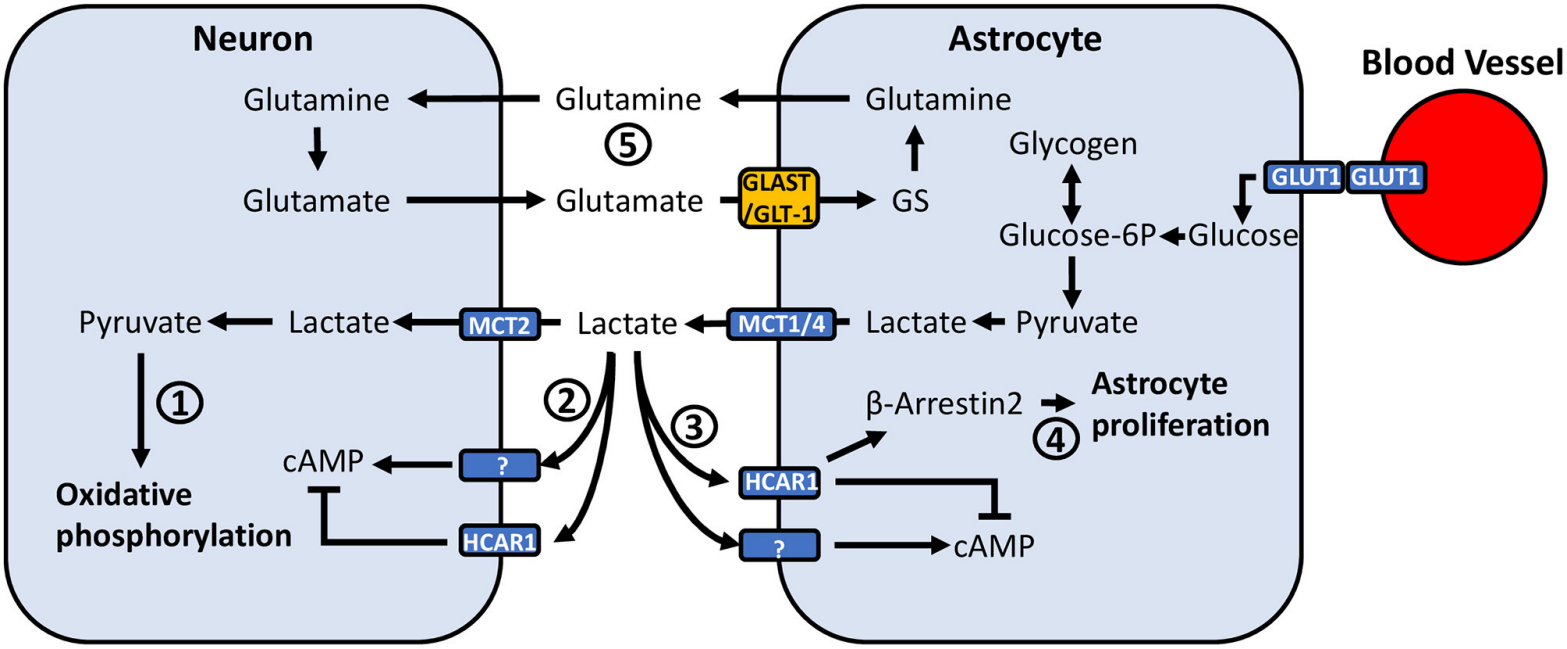
Potential mechanisms through which astrocyte-neuronal metabolic coupling and lactate release can mediate pain-related neuronal activity. (1) Lactate taken up by neurons via MCT2 is converted to pyruvate, where it can be used to generate ATP through oxidative phosphorylation. (2) Neurons express HCAR1, a G_i_-protein coupled receptor which inhibits adenylyl cyclase and reduces intracellular cAMP. Neurons also appear to express an unidentified lactate receptor which activates adenylyl cyclase and increases cAMP. (3) Similarly, astrocytes have been found to express HCAR1, however recent findings also suggest the presence of a lactate receptor which exerts opposite effects and activates rather than inhibits adenylyl cyclase. (4) Lactate binding to HCAR1 was recently associated with a non-G_i_-protein mechanism, acting through β-arrestin2-MAPK-signaling. The β-arrestin2-MAPK pathway has been associated with induction of astrocyte proliferation. (5) Glutamate released into the synaptic cleft is taken up by astrocytes via GLT-1 and GLAST. Astrocytic GS converts glutamate to glutamine, which is exported and taken up by neurons. Altered GLT-1/GLAST expression may affect synaptic transmission through dysregulation of extracellular glutamate concentrations.

Due to their close association with both neurons and blood vessels in the CNS, astrocytes are in a prime position to mediate energy supply to neurons (Figure 2). Astrocyte end feed processes make contact with blood vessels (172), allowing them to take up glucose from the blood via glucose transporter 1 (GLUT1) (173). Early culture studies demonstrated that astrocytes are able to accumulate glycogen in the presence of glucose (160). While both neurons and astrocytes express glycogen synthase (174, 175), a critical enzyme in glycogenesis, brain glycogen is predominantly localized to astrocytes rather than neurons (159). Astrocytes can break down glycogen to produce lactate (160), the majority of which is exported to the extracellular space rather than being consumed for energy (161, 162). In addition to findings that astrocytes can store glucose and export lactate, mechanisms by which lactate release is coupled to neuronal activity have been identified. Seminal work by Pellerin and Magistretti showed that glutamate uptake by astrocytes stimulates glucose uptake, glycolysis and lactate release, an effect dependent on extracellular Na+ and glutamate co-transport (176, 177). Further work identifying the expression of monocarboxylate transporters (MCT) in the brain, localization of different isoforms of lactate dehydrogenase in neurons and astrocytes, and that lactate was an efficient substrate for oxidative metabolism led to the proposal of the astrocyte neuron-lactate shuttle (178). This theory proposed that neuronal activity stimulates glycogenolysis and conversion of glucose to lactate, followed by its subsequent release from astrocytes and uptake by neurons to produce ATP during elevated activity (178).

Much of the work examining the consequences of lactate shuttling have been in the context of learning, memory, and reward associated learning in the brain. The formation of long-term memories has been shown to require hippocampal astrocytic lactate release during the initial acquisition period (179, 180). Indeed, training in an inhibitory avoidance task is accompanied by a rapid and sustained increase in extracellular lactate in the hippocampus (179), whereas inhibiting glycogen phosphorylase, the rate limiting enzyme in glycogenolysis, using 1,4-dideoxy-1,4,-imino-D-arabinitol 15 min prior to training abolishes the lactate rise, impairs LTP, and results in memory deficits (179, 180). In addition, inhibiting astrocytic lactate export by reducing MCT1 or MCT4 expression, or blocking neuronal lactate uptake by reducing MCT2 expression both result in memory impairments (179–181). The former can be rescued by lactate and its energetic equivalent pyruvate, but not by glucose (179, 180). Similar findings have been observed in drug-related memories involving the amygdala. In a conditioned place preference (CPP) paradigm, inhibiting glycogenolysis in the basolateral amygdala can prevent the acquisition of cocaine-induced place preference, transiently inhibit established CPP, and impair CPP following retrieval (182, 183). In both the hippocampus and amygdala, the induction of plasticity-related phosphorylation of cAMP response-element binding protein (CREB), cofilin, and ERK, is dependent on astrocytic glycogenolysis and L-lactate (179, 182). Notably, CREB is activated in the spinal cord and forebrain following tissue injury (184, 185), and transgenic over-expression of CREB in the forebrain enhances behavioral responses to the formalin model of temporary pain, and correspond with potentiated and more rapid development of pain hypersensitivity induced by nerve injury (186). Given that long term memory and chronic pain both involve persistent changes in synaptic activity, it is of interest whether astrocyte-neuronal lactate shuttling is involved in the pathogenesis of chronic pain.

## Initial Research on Astrocyte Lactate Export in Chronic Pain

Very few studies have directly examined the involvement of astrocyte-derived lactate in the context of pain. However, there is some evidence associating chronic pain with altered lactate dynamics in the CNS. One study found that MCT1 protein expression is elevated in the spinal dorsal horn 7 days after induction of chronic inflammatory pain by CFA (187), an observation that was also made in the hippocampus following inhibitory avoidance training (179). Another study in rats with chronic visceral hypersensitivity observed blunted activity-dependent lactate release in the ACC along with impaired decision making and synaptic plasticity (16). Additionally, molecular changes associated with long-term potentiation, such as the upregulation of pCREB or pERK in the spinal dorsal horn and the spinothalamic tract (10, 188–192), as well as in supraspinal regions such as the amygdala and anterior cingulate cortex (193–195) are also observed in chronic pain states. These parallels with memory-related synaptic plasticity suggest the possibility of other common mechanisms such as lactate shuttling in chronic pain. Recent work by Miyamoto et al. (196) showed that activating spinal astrocytes in mice using designer receptors exclusively activated by designer drugs (DREADDs) rapidly induces mechanical allodynia lasting for 10 h, accompanied by an increase in extracellular lactate levels. Accordingly, the broad MCT inhibitor α-Cyano-4-hydroxycinnamic acid (4-CIN) fully reversed this induced allodynia (196). They also found that intrathecal injections of 4-CIN could reduce mechanical allodynia, although not fully, in a partial sciatic nerve ligation model of neuropathic pain (196). At the time of drug administration, behavioral symptoms have already developed suggesting that inhibiting lactate shuttling can reduce pain hypersensitivity during the chronic phase. A study by a separate group also found that 4-CIN could partially alleviate mechanical allodynia during the chronic phase of a spinal-nerve ligation pain model (197). Hence, this initial evidence points to a possible role of spinal astrocytic lactate in maintaining pain hypersensitivity. This is a departure from findings in long term memory, where disrupting lactate beyond a certain window of time following either memory acquisition or retrieval has no effect on subsequent task performance (179, 182).

In addition to the work above, there have been studies which investigated pyruvate kinase M2 (PKM2), a glycolytic enzyme that catalyzes the dephosphorylation of phosphoenolpyruvate to pyruvate. Expression of PKM2 is elevated in the spinal dorsal horn along with lactate in both neuropathic and inflammatory pain models (198, 199). Inhibiting PKM2 reduces lactate elevations and partially alleviates mechanical allodynia and thermal hyperalgesia (198, 199). However, these studies also noted that inhibiting PKM2 prevented the enhanced expression of GFAP, TNFα, IL-1β, and phosphorylation of STAT3 amongst other proteins (198, 199). Indeed, PKM2 has been implicated to have functions beyond glycolysis, including activity as a protein kinase [see review by Dong et al. (200)]. But these effects may also relate to possible lactate signaling which will be discussed below. It is however, difficult to isolate the PKM2-mediated rise in lactate to astrocytes in these studies, as PKM2 expression was elevated in neurons and microglia as well (199).

## Lactate Signaling on Neuronal Excitability and Plasticity

Beyond acting as a metabolic substrate for ATP production, evidence for a signaling role of lactate complicates its effects in the CNS (Figure 2). The production of NADH by lactate in neurons has been shown to induce the expression of the plasticity-related immediate early genes *Arc, c-Fos* and *Zif268* (201). Additionally, neurons in the brain express the extracellular hydroxycarboxylic acid receptor 1 (HCAR1), a G_i_-protein coupled receptor which inhibits adenylyl cyclase and reduces intracellular cAMP (202, 203). L-lactate has been found to decrease the firing frequency of CA1 pyramidal cells in hippocampal slices, as well as in primary cortical neuron cultures via activation of HCAR1 (203, 204). Conversely, L-lactate can potentiate EPSCs, firing frequency, and spike probability of pyramidal cells in the CA3 region of hippocampal slices (205), and similarly increase firing frequency and neurotransmitter release in locus coeruleus slices (206) via a lactate receptor that has yet to be characterized (205–207). These effects are suggested to be metabolism-independent and mediated by extracellular signaling due to insensitivity to 4-CIN (204–206). These findings raise the possibility that population differences in the effects of extracellular lactate signaling may result in differential regulation of synaptic activity at various points along nociceptive signaling pathways.

## Lactate Signaling Implicated in Altering Astrocyte Function

Astrocytes may also respond to lactate (Figure 2), as they express HCAR1 (202, 208), which was recently associated with reducing glutamate-induced calcium influx via β-arrestin2-MAPK signaling (209). Kappa-opioid receptor activation of the β-arrestin2-ERK1/2 pathway induces astrocyte proliferation *in vitro* (210), and kappa-opioid receptor activation of p38-MAPK, which is also regulated by β-arrestin2 (211), has been implicated in astrocyte proliferation following sciatic nerve ligation in mice (212). Thus, lactate may promote astrocyte proliferation via a common intracellular signaling pathway, contributing to reactive astrogliosis. Recent findings have also identified that lactate can induce rises in intracellular cAMP and lactate via activation of adenylyl cyclase, suggesting the presence of another uncharacterized lactate receptor (213). cAMP is involved in a variety of signaling pathways and can modulate inducible nitric-oxide synthase activity (214, 215), cytokine release (216, 217), and astrocyte morphology (218).

Astrocytes in primary astroglial cultures incubated with 25 mM lactate show a significant increase in release of TNFα and IL-6 (219). Recent research in diabetic mice found that pyruvate dehydrogenase 2 (PDK2) expression was enhanced in hypothalamic astrocytes and contributed to inflammation (220). PDK2 phosphorylates and deactivates pyruvate dehydrogenase (221), shifting pyruvate metabolism to form lactate rather than enter the citric acid cycle. Genetically knocking out PDK2 reduced diabetes-induced elevation of lactate and induction of TNFα, IL-1β and IL-6, providing additional albeit indirect evidence for lactate-induced cytokine release (220). Furthermore, lactate uptake through MCT1 in oxygen and glucose deprived astrocyte cultures can upregulate expression of GFAP and phosphorylation of Akt and STAT3 (222); STAT3 is involved in reactive astrogliosis (223). These findings raise the possibility that aberrant lactate dynamics may facilitate astrocyte activation and their subsequent inflammatory cytokine profiles. However, given the expression of potentially two lactate receptors with opposing effects on adenylyl cyclase-cAMP signaling, the net effect of lactate on astrocytes in chronic pain is unclear.

Recent work by Bingul et al. (224) has provided interesting insight into long term lactate dynamics *in vivo* following LTP in the dentate gyrus of rats, with implications for lactate both as an energy substrate but also as a signaling molecule. Extracellular lactate levels change within seconds in response to acute electrical stimulation of the medial perforant pathway (224). The response is characterized by an initial dip in extracellular lactate followed by a larger overshoot, before returning to baseline (224). LTP induction causes a significant increase in the magnitude of the lactate dips and overshoots, starting at 24 h after potentiation, and induces an average chronic elevation of lactate concentrations that persists for 72 h (224). Whether these findings, in addition to lactate signaling described earlier, translates to CNS areas associated with mediating persistent pain has yet to be investigated, but they give rise to interesting possibilities. In the context of chronic pain, potentiated synaptic activity in the central nervous system may maintain persistently elevated extracellular lactate levels via activity-dependent release from astrocytes. Lactate, in addition to its metabolic role, is therefore in a position to mediate persistent effects on both astrocytes and neurons via extracellular receptor binding. However, lactate's functions as a signaling molecule in the CNS both under healthy and pathological conditions is poorly understood, requiring further research on how it may contribute to pathology.

## Conclusion

Astrocytes release a variety of metabolites and cytokines which have profound effects on neuronal activity. Pathology of the CNS is often associated with reactive astrogliosis, which is accompanied by altered release of these neuromodulatory substances. The findings presented here provide evidence for the involvement of altered astrocytic cytokine release in long term synaptic plasticity of central nociceptive pathways under chronic pain states. Gliotransmitters have also been implicated but given that microglia are involved in ATP and D-serine signaling, whether direct astrocyte-neuronal communication via altered gliotransmission contributes to the pathology of chronic pain is unclear. Lastly, the role of lactate derived from astrocytes as a neuronal energy substrate, and more recently as a signaling molecule in the CNS, has evolved significantly. However, very few studies have examined the involvement of lactate in the development and maintenance of chronic pain, presenting an exciting pathway for further research.

## Author Contributions

JT and MB drafted the review. JT formatted Figures 1, 2. JT and GD formulated the idea for the review and GD guided the research and writing process. All authors contributed to the article and approved the submitted version.

## Conflict of Interest

The authors declare that the research was conducted in the absence of any commercial or financial relationships that could be construed as a potential conflict of interest.
